# Analysis of Mice Lacking DNaseI Hypersensitive Sites at the 5′ End of the IgH Locus

**DOI:** 10.1371/journal.pone.0013992

**Published:** 2010-11-15

**Authors:** Thomas Perlot, Inka Pawlitzky, John P. Manis, Ali A. Zarrin, Peter H. Brodeur, Frederick W. Alt

**Affiliations:** 1 The Howard Hughes Medical Institute, The Children's Hospital, Immune Disease Institute, and Department of Genetics, Harvard Medical School, Boston, Massachusetts, United States of America; 2 Immunology Program, Sackler School of Graduate Biomedical Sciences, Tufts University School of Medicine, Boston, Massachusetts, United States of America; 3 Children's Hospital Boston and Joint Program in Transfusion Medicine, Harvard Medical School, Boston, Massachusetts, United States of America; National Institute on Aging, United States of America

## Abstract

The 5′ end of the IgH locus contains a cluster of DNaseI hypersensitive sites, one of which (HS1) was shown to be pro-B cell specific and to contain binding sites for the transcription factors PU.1, E2A, and Pax5. These data as well as the location of the hypersensitive sites at the 5′ border of the IgH locus suggested a possible regulatory function for these elements with respect to the IgH locus. To test this notion, we generated mice carrying targeted deletions of either the pro-B cell specific site HS1 or the whole cluster of DNaseI hypersensitive sites. Lymphocytes carrying these deletions appear to undergo normal development, and mutant B cells do not exhibit any obvious defects in V(D)J recombination, allelic exclusion, or class switch recombination. We conclude that deletion of these DNaseI hypersensitive sites does not have an obvious impact on the IgH locus or B cell development.

## Introduction

The variable region of an immunoglobulin heavy chain (IgH) is assembled from V (variable), D (diversity), and J (joining) gene segments that lie upstream of several IgH constant (C) region exons in a process called V(D)J recombination [1]. The mouse IgH locus contains large numbers of V_H_ segments and multiple D and J_H_ segments but an individual IgH V(D)J exon is assembled from only one V_H_, one D, and one J_H_ segment.

V(D)J recombination of the IgH locus takes place in pro-B cells in an ordered way such that D to J_H_ recombination precedes V_H_ to DJ_H_ recombination [2]. In this regard, activation of the IgH locus is thought to progress in a stepwise manner [3]. D to J_H_ rearrangement efficiently occurs on both alleles, however, allelic exclusion ensures that V_H_ to DJ_H_ recombination results in expression of a functional heavy chain (HC) from only one of the two alleles [4].

Mature B-cells can undergo further alterations of their HCs. IgH class switch recombination (CSR) causes expression of different immunoglobulin isotypes which confer different effector functions. During this recombination process one of several sets of downstream C_H_ exons replaces the Cµ exons and the intervening sequence is deleted from the chromosome, which results in expression of a new C region without changing the specificity of the IgH variable region [5].

A large effort has been made to elucidate mechanisms of IgH locus regulation and a number of cis-regulatory elements have been described and characterized. The IgH intronic enhancer (Eµ) resides in the J_H_ – C_H_ intron and was shown to be necessary for efficient V(D)J recombination by promoting both D to J_H_ and V_H_ to DJ_H_ recombination [6], [7]. Downstream of the C_H_ genes at the very 3′ end of the IgH locus a cluster of DNaseI hypersensitive sites was described, termed 3′ IgH regulatory region (3′IgH RR). So far two main functions have been assigned to this regulatory region: the 3′IgH RR plays an important role in promoting CSR to most IgH isotypes, and the 3′IgH RR was shown to be necessary for high level expression of the functionally assembled HC gene from the promoter 5′ of the V_H_DJ_H_ exon [8].

An additional potential regulatory region was identified at the 5′ end of the IgH locus, consisting of four DNaseI hypersensitive sites [9]. One of these sites, HS1, was shown to be pro-B cell specific, the stage during which IgH V(D)J recombination takes place, and was suggested to include binding sites for the transcription factors PU.1, Pax5 and E2A [9]. These observations led to the suggestion that this region might represent a new regulatory region for IgH rearrangements. In this regard, the 5′ end of the IgH locus is an attractive location for a regulatory element because it would not be deleted during the course of V(D)J recombination, and it might explain control of several unresolved phenomena in the IgH locus. Among these is the regulation of V_H_ germline transcripts as so far no cis-regulatory element has been identified that controls activity of the bulk of unrearranged V_H_ promoters. Furthermore, it is not known how it is achieved that proximal and distal V_H_ segments are activated independently or why usage of distal versus proximal V_H_ gene families varies significantly.

Here we report the targeted deletion of the pro-B cell specific 5′IgH HS1 as well as combined deletion of HS1, HS2, HS3a,b in mice. We analyzed potential implications on B cell development, V(D)J recombination, and IgH CSR.

## Methods

### Targeted deletion of 5′IgH DNaseI hypersensitive sites in ES cells and generation of mutant mice

**Figure 1:**
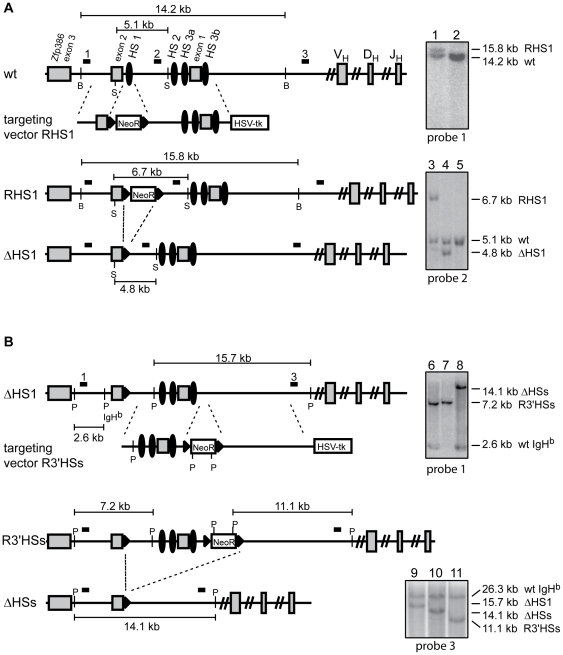
Targeting strategy for the generation of the *RHS1*, *ΔHS1*, and *ΔHSs* alleles. (A) the wildtype (wt) IgH locus and its 5′ flanking region are shown. V_H_, D_H_, J_H_ indicate representative IgH V, D, and J segments. Exons 1, 2, and 3 of *Zfp386* are shown as grey rectangles, DNaseI hypersensitive sites HS1, HS2, HS3a, and HS3b are shown as black ovals. Correct targeting events of the RHS1 targeting vector were identified by Southern blotting on BglII digested ES cell DNA using probe 1, which results in a 15.8 kb band (lane 1) in addition to the 14.2 kb wildtype band (lane 2). Cre – loxP (black triangles) mediated deletion of the PGK-Neo^R^ cassette (NeoR) from the *RHS1* allele results in the *ΔHS1* allele. Deletions were identified by Southern analysis of SacI digested DNA utilizing probe 2. A targeted clone before Cre – mediated deletion exhibits a 6.7 kb *RHS1* band and a 5.1 kb wildtype band (lane 3). Upon deletion of the PGK-Neo^R^ cassette, a 4.8 kb *ΔHS1* band and a 5.1 kb wildtype band are visible (lane 4). Lane 5 shows untargeted wildtype DNA. (B) The *ΔHS1* allele was targeted with the R3′HSs targeting vector to introduce a PGK-Neo^R^ cassette flanked by loxP sites. Correct targeting events were confirmed by Southern blotting on SphI digested ES cell DNA with probe 1, resulting in a 2.6 kb band for the wildtype IgH^b^ allele and a 7.2 kb band for *R3*′*HSs*, the targeted IgH^a^ allele (lane 6). Cre – mediated recombination between the first and the third loxP site generates the *ΔHSs* allele (14.1 kb, lane 8). Homozygous R3′HSs ES cells were generated under increasing concentrations of G418, resulting in a single 7.2 kb R3′HSs band (lane 7). Southern analysis on SphI digested DNA with probe 3 confirms correct targeting events of the R3′HSs targeting vector. *ΔHS1* ES cells exhibit a 15.7 kb *ΔHS1* band and a 26.3 kb band for the wildtype IgH^b^ allele (lane 9), *R3*′*HSs* ES cells show a 11.1 kb *R3*′*HSs* band and a 26.3 kb band for the wildtype IgH^b^ allele (lane 11), *ΔHSs* ES cells are identified by the presence of a 14.1 kb *ΔHSs* band in addition to the 26.3 kb wildtype IgH^b^ allele (lane 10). All targeting events occured on the IgH^a^ allele, whereas the IgH^b^ allele remained in wildtype configuration. Drawings not to scale. B - BglII; S - SacI; P - SphI.

All mouse were handled in strict accordance with good animal practice as defined by the relevant national and/or local animal welfare bodies, and all animal work was approved by Animal Research of Children's Hospital Boston (Protocol # 08 11 1253R). The RHS1 targeting vector was assembled in pLNTK [10]. As a 5′ homology arm a 2.2 kb PCR product was generated with primers 5′ GTCGACGGATTTAGGAGGATACACAAC 3′ and 5′ GTCGACCTTGGATAACACAGAACTCTG 3′ containing a SalI site at their 5′ ends, which facilitate cloning of the PCR product into the SalI site of pLNTK. As a 3′ homology arm a 7.3 kb AatII – ApaI fragment was blunt end cloned into the XhoI site of pLNTK. The R3′HSs targeting vector was generated by blunt end cloning a 4.4 kb EcoRI fragment into the SalI site of pLNTK as a 5′ homology arm, and a 7.0 kb KpnI fragment into the XhoI site as the 3′ homology arm. Correct targeting events and cre – loxP deletion events were confirmed by Southern blotting (Fig 1). Probe 1 is a 830 bp PCR product amplified with primers 5′ GCTCATGTACCAATCTGCACTCAC 3′ and 5′ CACTGTGACCTCCATCTTATGTCTG 3′. Probe 2 is a 1.2 kb PstI – EcoRI fragment 5′ of HS2. Probe 3 is a 0.8 kb PstI – XbaI fragment about 11 kb 3′ of HS3b. To confirm single integration of the targeting vectors a 525 bp Neo^R^ probe was used, amplified with primers 5′ GCAGCCATATGGGATCGGC 3′ and 5′ GTTCGGCTGGCGCGAGCCCC 3′.

EF1 heterozygous IgH^a/b^ embryonic stem (ES) cells, generated in the Alt laboratory, were transfected with PvuI linearized RHS1 targeting vector to obtain *RHS1/+* ES cells. To obtain *ΔHS1/+* ES cells, the PGK-Neo^R^ cassette was deleted by applying a Cre – expressing adenovirus vector. *ΔHS1/+* ES cells were transfected with PvuI linearized R3′HSs targeting vector to obtain *R3*′*HSs/+* ES cells. *R3*′*HSs/+* ES cells were selected for homozygocity of the targeted allele through increasing concentration of G418 to obtain *R3*′*HSs/R3*′*HSs* ES cells. Cre – loxP mediated deletion of the PGK-Neo^R^ cassette resulted in *ΔHSs/ΔHSs* ES cells. Targeted ES cells were injected into *Rag2-/-* blastocysts to obtain RDBC chimeras [11] or into C57BL/6 blastocysts to obtain chimeras that could be crossed to 129Sv mice to achieve germline transmission of the targeted allele.

### B cell hybridomas

CD43^-^ splenocytes were isolated by MACS, stimulated with LPS (20 µg/ml), and fused to NS-1 plasmacytoma cells (TIB-18, ATCC) as described previously [12]. IgH V(D)J rearrangement status was analyzed by Southern blotting of EcoRI digested genomic DNA of clonal hybridomas with three different probes, a 1.6 kb HindIII - EcoRI fragment 3′ of J_H_4, a 0.38 kb SacI - ApaI fragment 3′ of D_H_Q52, and a 0.75 kb PCR product 5′ of D_H_FL16.1 generated with oligonucleotides 5′ GAACAGCAACCCTTGACTGACTCTG 3′ and 5′ GATTGGTTCTTATGGAATGGGTGG 3′.

### PCR assay for V(D)J rearrangements

Pro-B cells (IgM^-^ B220^+^ CD43^hi^), pre B-cells (IgM^-^ B220^+^ CD43^lo^), and double positive T-cells (B220^-^ CD4^+^ CD8^+^) were isolated by FACS on a FACSAria (BD Biosciences) and genomic DNA was extracted. 50 ng DNA or 5-fold dilutions were analyzed by PCR for D_H_–J_H_, V_H_-DJ_H_, V_κ_–J_κ_, and V_λ_–J_λ_ rearrangements with primers listed in Table S1. Input DNA amounts were normalized upon PCR amplification within DLG5. PCR was performed at 95°C for 4′, 30 cycles of 95°C for 30″, 60°C for 90″, and 72°C for 2′, followed by 72°C for 5′. PCR products were transferred from ethidium bromide gels to nylon membranes and visualized with end labeled oligonucleotide probes (Table S1). CDR 3 lengths were generated from IgH VDH rearrangements from mature B cells using oligonucleotides for V558 and JH4 rearrangements. PCR fragments were amplified using iProof (Bio-Rad) polymerase and cloned into Zero Blunt Topo vectors (Invitrogen), and sequenced.

### IgH class switch recombination assay

CD43^-^ splenocytes were isolated by MACS, cultured with LPS or IL4/αCD40, and analyzed by flow cytometry as described previously [13].

### RT-PCR analysis

RNA was extracted using TriPure Isolation Reagent (Roche). 200 ng–1 µg of total RNA was reverse transcribed for one hour at 50°C using random hexamers (Roche) and Superscript III (Invitrogen) reverse transcriptase. PCR was performed at 94°C for 4′, 30–39 cycles of 94°C for 30″, annealing temperature (Table S1) for 30″, 72°C for 30″, followed by 72°C for 5′. cDNA input amount was normalized upon PCR amplification of β-actin cDNA. PCR products were visualized on ethidium bromide gels and/or subsequently transferred to nylon membranes and visualized with end labeled oligonucleotide probes (Table S1).

### Flow cytometry and cell sorting

Single cell suspensions from spleen, thymus, or bone marrow were stained in PBS 2% FBS with various antibodies: FITC-αIgM, PE-Cy5-αB220, PE-αCD8a, PE-αCD43, FITC-αLy9.1, APC-αIgM, APC-Cy7-αB220 (BD Pharmingen), PE-αAA4.1, FITC-αCD4 (eBioscience). FACS analysis was performed on a FACSCalibur (BD Biosciences) and a FACSAria (BD Biosciences) apparatus. Cell sorts were performed on a FACSAria (BD Biosciences) apparatus.

## Results

### Generation of mice with targeted deletion of 5′IgH DNaseI hypersensitive sites

To determine the *in vivo* function of the cluster of DNaseI hypersensitive sites described at the 5′ end of the IgH locus [9] we first replaced a ∼340 bp BccI – AatII fragment, harboring HS1, with a loxP flanked PGK-Neo^R^ cassette. All targeting experiments were performed in heterozygous IgH^a/b^ EF1 ES cells which have the advantage that IgH^a^ (129 strain) and IgH^b^ (C57BL/6 strain) alleles can be distinguished by antibodies against the different allotypes or by detection of restriction fragment length polymorphisms (RFLP). Targeting vector homology arms were cloned from 129 strain genomic DNA, resulting in correct targeting events only on the IgH^a^ allele. In heterozygous targeted ES cells, the IgH^b^ allele always remained in the untargeted wildtype configuration.

Targetings were performed with the RHS1 targeting vector (Fig. 1A) to obtain the *RHS1* allele and, upon cre/loxP deletion, the *ΔHS1* allele. Correct targeting events (Fig1) and single integration of the targeting vector (Figure S1) were confirmed by Southern blotting. Subsequently, targeted ES cells were injected into *Rag2-/-* blastocysts to obtain Rag-deficient blastocyst complementation (RDBC) chimeras, and into wildtype blastocyts to generate mice that carry the *RHS1* or *ΔHS1* allele in their germline. In order to delete all four hypersensitivity sites (HS1, HS2, HS3a, and HS3b), ES cells containing the *ΔHS1* allele were targeted with the R3′HSs targeting vector to obtain the *R3*′*HSs* allele (Fig. 1B). Cre/loxP recombination between the loxP site originating from the *ΔHS1* allele and the loxP site 3′ of the PGK-Neo^R^ cassette results in the replacement of a 8.9 kb region, harboring all described 5′IgH DNaseI hypersensitive sites, with a single loxP site, referred to as the *ΔHSs* allele. Germline transmission could not be achieved for either of the *R3*′*HSs* or *ΔHSs* heterozygous ES cell lines. Therefore, we placed ES cells containing the *R3*′*HSs* allele under increasing concentrations of G418 to select for homozygous mutant ES cells. The homozygous mutant ES cells were subsequently subjected to cre/loxP recombination to delete the Neo^r^ gene and generate ES cells homozygous for the *ΔHSs* allele. The homozygous mutant *ΔHSs* ES cells were injected into *Rag2^-/-^* blastocysts, and chimeras generated by RDBC and lymphocytes were analyzed.

### Development of homozygous *RHS1*, *ΔHS1*, and *ΔHSs* lymphocytes

**Figure 2:**
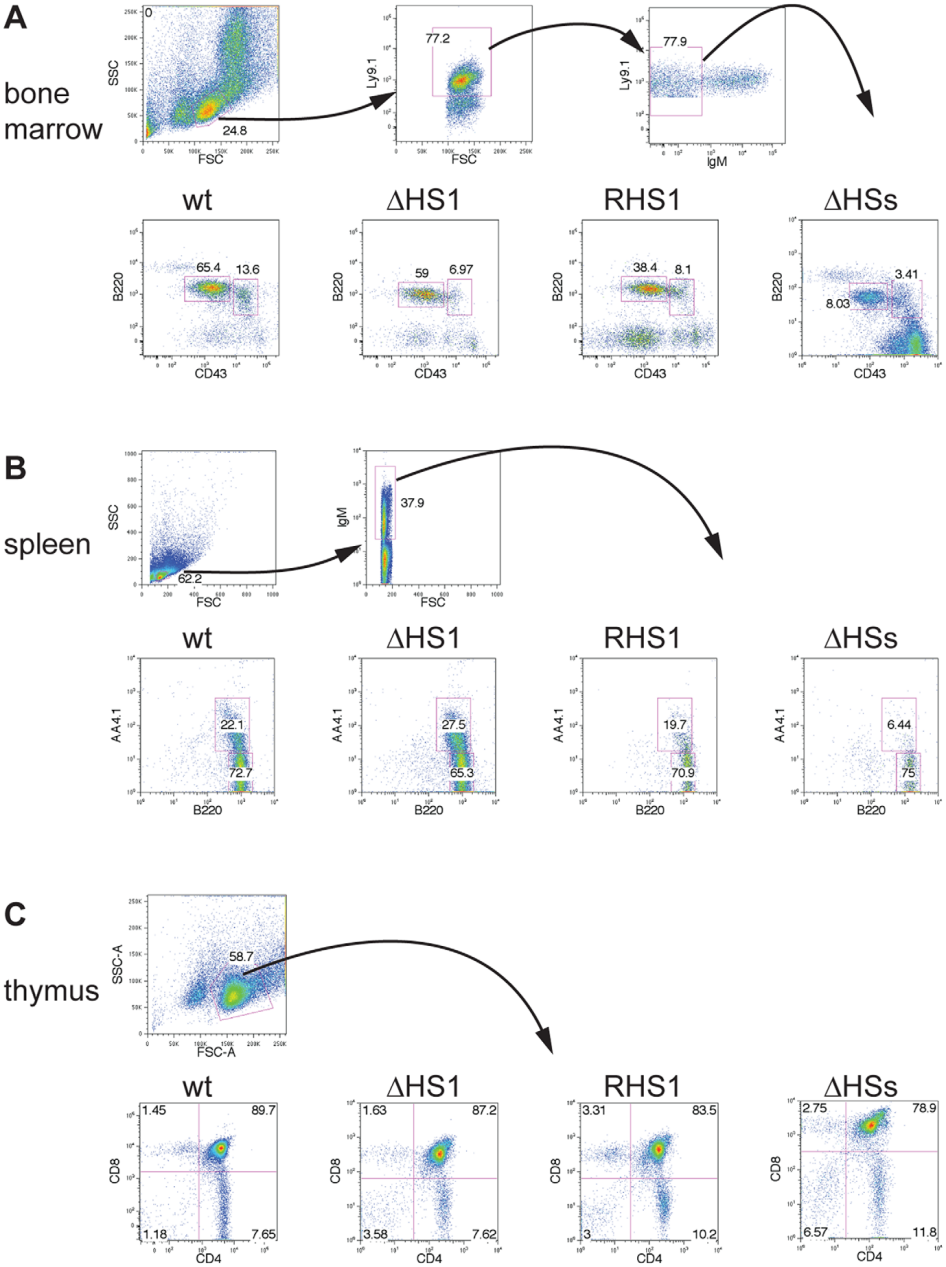
Development of homozygous *ΔHS1*, *RHS1*, and *ΔHSs* lymphocytes. (A) Bone marrow from wildtype (wt), homozygous *ΔHS1*, homozygous *RHS1* mice, and RDBC chimeras generated from homozygous *ΔHSs* ES cells was subjected to FACS analysis. Gates were set on the lymphocyte population, Ly9.1 positive population, and on IgM negative population (upper three blots, left to right) to analyze pro-B cell (IgM^-^ B220^+^ CD43^hi^) and pre-B cell (IgM^-^ B220^+^ CD43^lo^) populations (lower blots). (B) FACS analysis of splenocytes from wildtype (wt), homozygous *ΔHS1*, homozygous *RHS1* mice, and RDBC chimeras generated from homozygous *ΔHSs* ES cells. Gates were set on the lymphocyte population and on the IgM positive population (upper two blots, left to right) to analyze transitional B-cell (IgM^+^ B220^+^ AA4.1^+^) and mature B-cell (IgM^+^ B220^+^ AA4.1^-^) populations (lower blots). (C) FACS analysis of thymocytes gated on the lymphocyte population (upper blot) from wildtype (wt), homozygous *ΔHS1*, homozygous *RHS1* mice, and RDBC chimeras generated from homozygous *ΔHSs* ES cells (lower blots).

Lymphocytes of different developmental stages can be identified by FACS analysis of cells from lymphoid tissues such as bone marrow, thymus, or spleen. We analyzed 8 week old wildtype mice, homozygous *RHS1*, and homozygous *ΔHS1* mice that carry the mutant alleles in their germline, as well as lymphocytes from RDBC chimeras generated from homozygous *ΔHSs* ES cells (Fig. 2). In wildtype bone marrow, pro-B cells can be identified as IgM^-^ B220^+^ CD43^hi^ and pre-B cells as IgM^-^ B220^+^ CD43^lo^ cells, respectively. Defects in B-cell development can be revealed by the increase or decrease of certain lymphocyte populations. In this regard, impaired IgH V(D)J recombination leads to an accumulation of pro-B cells and to reduced numbers of pre-B cells [7]. We performed FACS analyses of bone marrow from three mice of each genotype to measure the percentage of pro- and pre-B cells in the lymphocyte gate. These analyses revealed the average percentage (± standard deviation) of pro-B and pre-B cells, respectively of B220^+^/CD43^+^ events in the total lymphocyte gate were 14±2 and 50±20 for wildtype, 9±3 and 56±6 for *ΔHS1*, and 9±3 and 42±11 for *RHS1* mice (Fig. 2A). Thus, there were no obvious differences in early B-cell development in wildtype and mutant mice. However we cannot exclude minor developmental defects not readily detectable by such analyses. Homozygous mutant *ΔHSs* bone marrow cells were analyzed in a similar fashion, but only Ly9.1^+^ cells were included in the analysis. Ly9.1 is exclusively expressed on cells derived from the *Δ*HSs ES cells but not on cells derived from the *Rag2-/-* blastocyst. The presence of a large compartment of blastocyst derived Rag-deficient pro-B cells in the bone marrow can interfere with development of ES cell derived B-lymphocytes. However, FACS analysis of *ΔHSs* bone marrow B cells indicated the presence of both pro- and pre-B cells and did not suggest a block in B-cell development (Fig. 2A).

Next we analyzed spleens for IgM^+^ B220^+^ AA4.1^+^ transitional B-cells and IgM^+^ B220^+^ AA4.1^-^ mature B-cells (Fig. 2B). In homozygous *RHS1*, and homozygous *ΔHS1* mice transitional (19.7%–27.5%) and mature (65.3%–72.7%) B-cell compartments similar to wildtype were identified; whereas, in spleens from RDBC chimeras generated from homozygous *ΔHSs* ES cells strongly reduced numbers of transitional B-cells were observed (6.44%). This reduction in the transitional B-cell compartment compared to the mature B-cell compartment (75%) might be due to overall reduced numbers of developing B cells in the obtained RDBC chimeras and to the accumulation of mature B-cells in the periphery of these mice and not to a defect in B cell development. Finally, we observed normal development of T-lymphocytes in the thymi of wildtype, homozygous *RHS1*, and homozygous *ΔHS1* mice as well as RDBC chimeras generated from homozygous *ΔHSs* ES cells (Fig. 2C).

### The *ΔHS1*, *RHS1*, and *ΔHSs* alleles show no significant defect in V(D)J recombination

**Figure 3:**
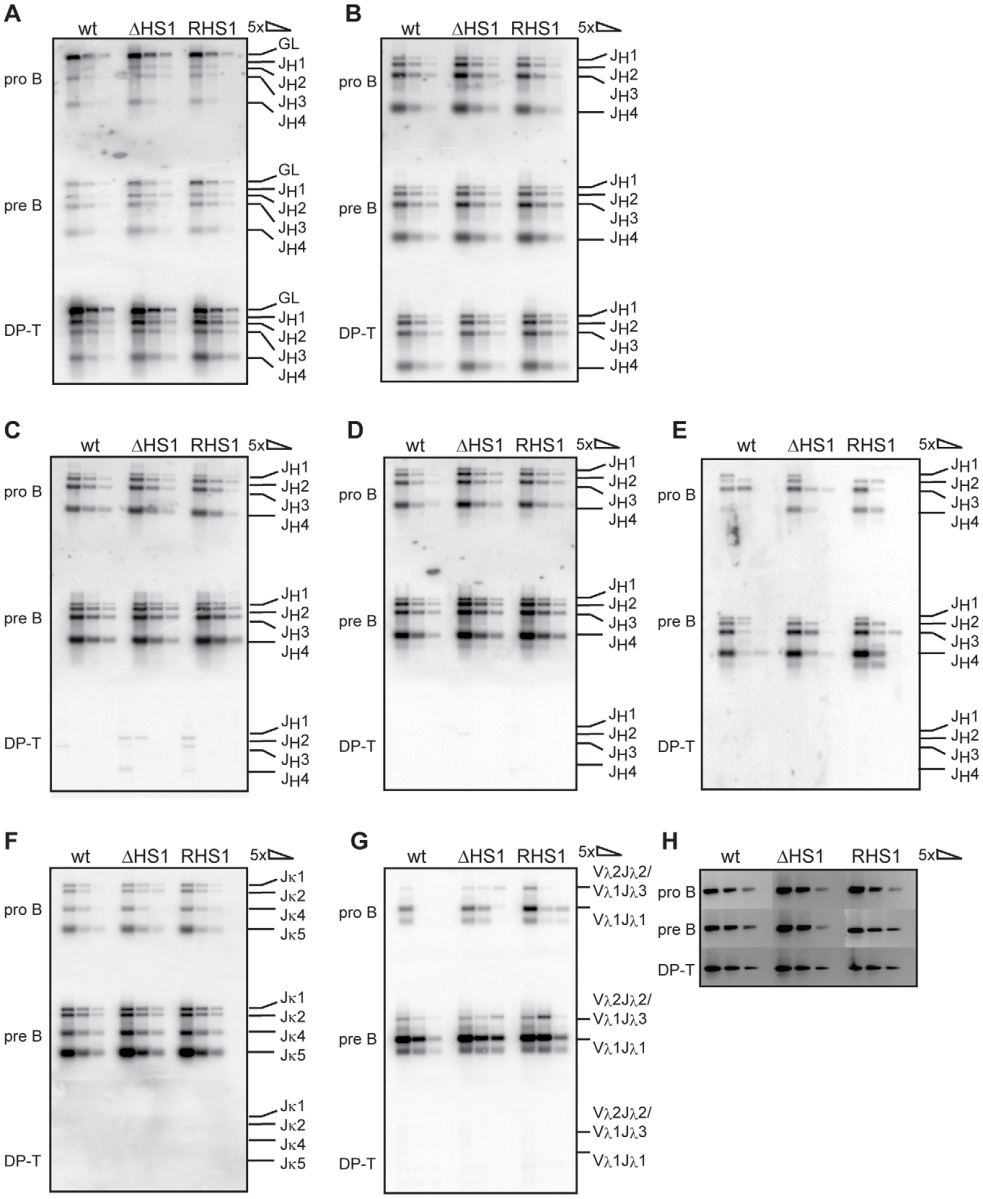
V(D)J recombination in *ΔHS1* and *RHS1* mice. Pro-B cells, pre-B cells and double positive (DP) T-cells from wildtype 129 mice, from homozygous *ΔHS1*, and homozygous *RHS1* mice were sorted by FACS. 5-fold dilutions of genomic DNA were subjected to PCR analysis. IgH V(D)J recombination efficiencies were assessed using a reverse primer downstream of J_H_4 and a forward primer recognizing DQ52 (A), DSPs (B), the V_H_7183 family (C), the V_H_J558 family (D), or the V_H_J558.55 segment (E). Rearrangements can occur to J_H_1, J_H_2, J_H_3, or J_H_4 as indicated. GL indicates PCR product from germline configuration. Igκ rearrangements were quantified (F), rearrangements can occur to J_κ_1, J_κ_2, J_κ_4, or J_κ_5 as indicated. Igλ rearrangement efficiency was analyzed (G). Bands correspond to V_λ_2 - J_λ_2, V_λ_1 - J_λ_3, or V_λ_1 - J_λ_1 rearrangements as indicated. DNA input was normalized to *DLG5* PCR products (H).

The data indicating that HS1 is pro-B cell specific and contains binding sites for the transcription factors PU.1, Pax5, and E2A led to the suggestion that HS1 could be involved in regulation of V(D)J recombination at the IgH locus [9]. We utilized a PCR based assay to assess V(D)J recombination efficiencies in developing lymphocytes from mice with homozygous deletion of HS1. FACS-sorted pro-B cells (IgM^-^ B220^+^ CD43^hi^) and pre-B cells (IgM^-^ B220^+^ CD43^lo^) from bone marrow and double positive (DP) T-cells (B220^-^ CD4^+^ CD8^+^) from thymus were analyzed for D to J_H_, V_H_ to DJ_H_, V_κ_ to J_κ_ and V_λ_ to J_λ_ rearrangements. Intensities of PCR bands for D_H_Q52 to J_H_ (Fig. 3A) and DSP to J_H_ rearrangements (Fig. 3B) were comparable in pro-B cells, pre-B cells, and DP T-cells from wildtype, homozygous *RHS1*, and homozygous *ΔHS1* mice indicating that deletion of the pro-B cell specific HS1 site does not detectably affect the D to J_H_ recombination step. DNA input amounts were normalized to the presence of a genomic sequence within the murine *DLG5* gene (Fig. 3H).

It was speculated that HS1 might regulate the differential activation of distal versus proximal V_H_ families [9]; therefore, we analyzed the rearrangement efficiencies of the proximal V_H_7183 family (Fig. 3C), the distal V_H_J558 familiy (Fig. 3D), and the distal most V_H_ segment V_H_J558.55 (Fig. 3E). We found that pro-B cells and pre-B cells from wildtype, homozygous RHS1, and homozygous ΔHS1 mice rearrange the proximal V_H_7183 family at similar levels (Fig. 3C). Also, the distal family V_H_J558 (Fig. 3D) as well as the distal most V_H_ segment V_H_J558.55 (Fig. 3E) rearranged at comparable efficiencies in pro-B cells and pre-B cells from the three different genotypes. V_H_ to DJ_H_ recombination was absent in DP T-cells from wildtype, homozygous *RHS1*, and homozygous *ΔHS1* mice as the V_H_ to DJ_H_ recombination step is restricted to the B-lineage (Fig 3C, D, E). These data show that HS1 is not necessary for rendering the distal part of the V_H_ cluster accessible and, therefore, suggest that HS1 does not play a major role in regulation of usage or accessibility of distal versus proximal V_H_ families.

Recently, it has been shown that IgH and Igκ loci can colocalize during B-cell development, mainly at the pre-B cell stage, and it was suggested that this colocalization induces decontraction of the IgH locus [14]. We therefore performed an assay to evaluate Igκ (Fig. 3F) and Igλ (Fig. 3G) V(D)J recombination efficiencies. Both Igκ and Igλ loci show similar V(D)J recombination levels in the analyzed developing B cells from wildtype, homozygous *RHS1*, and homozygous *ΔHS1* mice, while light chain rearrangements were absent in DP T-cells from the three different genotypes. Therefore, we conclude that deletion of HS1 does not markedly affect Ig light chain gene rearrangements.

As an independent method to evaluate D to J_H_ and V_H_ to DJ_H_ recombination efficiencies, we generated clonal hybridoma lines from splenic B-cells of IgH^a/b^ heterozygous *RHS1*, *ΔHS1* mice carrying the mutant allele in their germline and of RDBC chimeras generated from heterozygous *ΔHSs* ES cells (Table 1). In each case the IgH^a^ allele was the mutant allele while the IgH^b^ allele was the wildtype allele. In splenic B-cells, one allele exists as a functional V_H_DJ_H_ rearrangement, while the second allele can either be in germline configuration, or it exists as a DJ_H_ or an nonproductive V_H_DJ_H_ rearrangement. The rearrangement status of the second IgH allele was assessed by Southern blot analysis. Consequently, hybridomas expressing the mutant IgH^a^ allele can be analyzed for rearrangement efficiency of the wildtype IgH^b^ allele, and vice versa, in hybridomas expressing the wildtype IgH^b^ allele, the rearrangement status of the mutant IgH^a^ allele can be assessed.

**Table 1:** *ΔHS1*, *RHS1*, and *ΔHSs* hybridoma analysis.

		DJ	VDJ-
*ΔHS1*	IgM^a+^	66 (61%)	43 (39%)
*ΔHS1*	IgM^b+^	52 (57%)	39 (43%)
*RHS1*	IgM^a+^	62 (56%)	49 (44%)
*RHS1*	IgM^b+^	45 (52%)	41 (48%)
*ΔHSs*	IgM^a+^	51 (61%)	32 (39%)
*ΔHSs*	IgM^b+^	55 (69%)	25 (31%)

Hybridomas were generated from heterozygous IgM^a/b^ *ΔHS1*, *RHS1*, and *ΔHSs* splenic B-cells. In each case, IgM^b^ is the wildtype allele and IgM^a^ is the mutant allele. IgM^a^ expressing hybridomas (IgM^a+^) and IgM^b^ expressing hybridomas (IgM^b+^) of each genotype were analyzed for the rearrangement status of their nonproductive allele. Numbers for D to J_H_ rearranged alleles (DJ) and nonproductive V_H_ to DJ_H_ rearranged alleles (VDJ-) are shown.

Wildtype B cells undergo D to J_H_ rearrangements on both alleles; but still, consistent with earlier studies, about 5% of hybridomas harbor an IgH allele in germline configuration which presumably originates from tripartite fusions involving non B-cells [12] (not shown). The number of mutant alleles in germline configuration was not increased compared to wildtype indicating that *RHS1*, *ΔHS1*, and *ΔHSs* alleles can undergo efficient D to J_H_ recombination (not shown). In 50–60% of wildtype B-cells the nonproductive allele is in DJ_H_ configuration; whereas in 40–50% the nonproductive allele is in V_H_DJ_H_ configuration [15]. An increased percentage of DJ_H_ alleles could indicate less efficient V_H_ to DJ_H_ recombination: in contrast, an increased percentage of V_H_DJ_H_ alleles might indicate a break in allelic exclusion. IgM^a^ expressing hybridomas generated from B-cells heterozygous for *RHS1*, *ΔHS1*, and *ΔHSs* were analyzed for their rearrangement status of the wildtype IgM^b^ allele and show ratios of DJ_H_ (56%–61%), and V_H_DJ_H_ alleles (39%–44%) in the expected range (Table 1). IgM^b^ expressing hybridomas were analyzed for the rearrangement status of their mutant IgM^a^ allele. *RHS1*, *ΔHS1*, and *ΔHSs* alleles do not show significantly increased or decreased (Fisher's exact test) rearrangement ratios compared to wt alleles, as 52%–69% of mutant alleles were in DJ_H_ configuration while 31%–48% were in V_H_DJ_H_ configuration.

**Figure 4:**
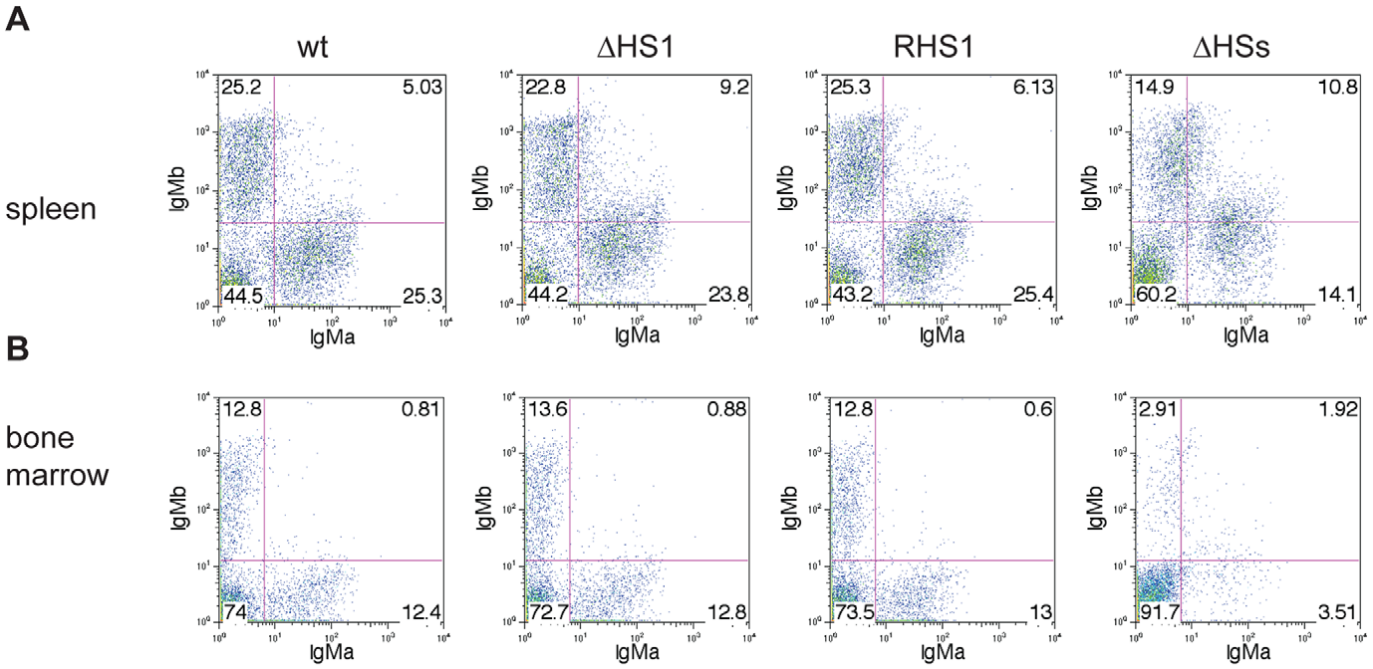
IgM^a^ versus IgM^b^ expression in *ΔHS1*, *RHS1*, and *ΔHSs* B cells. Heterozygous IgM^a/b^ B-cells from spleen (A) and bone marrow (B) of 129 wildtype (wt) or *ΔHS1*, *RHS1*, and *ΔHSs* RDBC chimeras were analyzed IgM^a^ and IgM^b^ expression. In *ΔHS1*, *RHS1*, and *ΔHSs* B-cells the IgM^b^ allele is in wildtype configuration whereas the IgM^a^ allele is the mutant allele.

FACS analysis was performed on B-cells from spleens (Fig. 4A) and bone marrow (Fig. 4B) of RDBC chimeras generated from heterozygous *RHS1*, *ΔHS1*, and *ΔHSs* ES cells. IgM^a^ expressing populations, representing the targeted allele, and IgM^b^ expressing populations, representing the wildtype allele, were of similar size both in bone marrow and in spleen from *RHS1*, *ΔHS1*, and *ΔHSs* chimeras, suggesting that the *RHS1*, *ΔHS1*, and *ΔHSs* alleles can undergo V(D)J recombination at the IgH locus at similar efficiencies as wildtype alleles.

### The *ΔHS1*, *RHS1*, and *ΔHSs* alleles do not affect allelic exclusion

FACS analysis of wt B cells from spleen (Fig. 4A) and bone marrow (Fig. 4B) shows distinct populations of similar size for B cells that are single positive for either IgH^a^ or IgH^b^, but intact allelic exclusion prevents the appearance of an obvious IgH^a^, IgH^b^ double producing population. Similarly, RDBC chimeras generated from heterozygous *RHS1*, *ΔHS1*, and *ΔHSs* ES cells exhibited IgH^a^ or IgH^b^ single positive B-cell populations of similar size in spleen (Fig. 4A) and bone marrow (Fig. 4B) but no IgH^a^, IgH^b^ double producing population. These data indicate that the deleted sequences of the targeted alleles do not contain a regulatory element that is necessary for implementation of allelic exclusion. Furthermore, data from hybridoma analysis (Tab. 1) support this notion as in the case of a break in allelic exclusion increased numbers of hybridomas with V_H_ to DJ_H_ rearrangements on both alleles would be expected. Such an increase compared to wildtype alleles could not be observed (Tab. 1), which indicates intact allelic exclusion of *RHS1*, *ΔHS1*, and *ΔHSs* alleles.

### The 5′IgH DNaseI hypersensitive sites are not required for efficient class switch recombination

**Figure 5:**
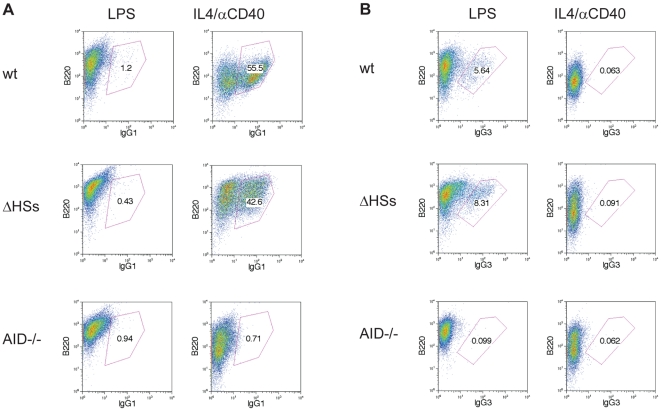
Ig class switch recombination in absence of the 5′IgH DNaseI hypersensitive sites. MACS purified splenic B-cells were stimulated in culture with LPS or IL4+ αCD40 as indicated. FACS analysis shows B-cells that underwent CSR as B220^+^ IgG1^+^ or B220^+^ IgG3^+^ cells, respectively. *AID* ^-/-^ B-cells served as negative controls, wildtype (wt) 129 B-cells as positive controls. Homozygous *ΔHSs* B-cells were isolated from RDBC chimeras.

To assess a potential effect of the 5′IgH DNaseI hypersensitive sites on CSR, B-cells were stimulated to undergo CSR and analyzed by FACS (Fig. 5). Stimulation with LPS induces IgH isotype switching to γ3, while stimulation with IL4+ αCD40 promotes switching to γ1. B-cells from AID-/- mice served as negative controls, while wildtype B-cells represented a positive control and therefore switched to the appropriate isotypes under LPS or IL4+ αCD40 stimulation. CSR in homozygous *ΔHSs* B-cells occurs at similar levels as in wildtype B-cells implying that the cluster of 5′IgH DNaseI hypersensitive sites is not required for efficient CSR to γ1 (Fig. 5A) and γ3 (Fig. 5B).

### Complex phenotypes without an obvious relation to the IgH locus in *ΔHS1* mice

**Figure 6:**
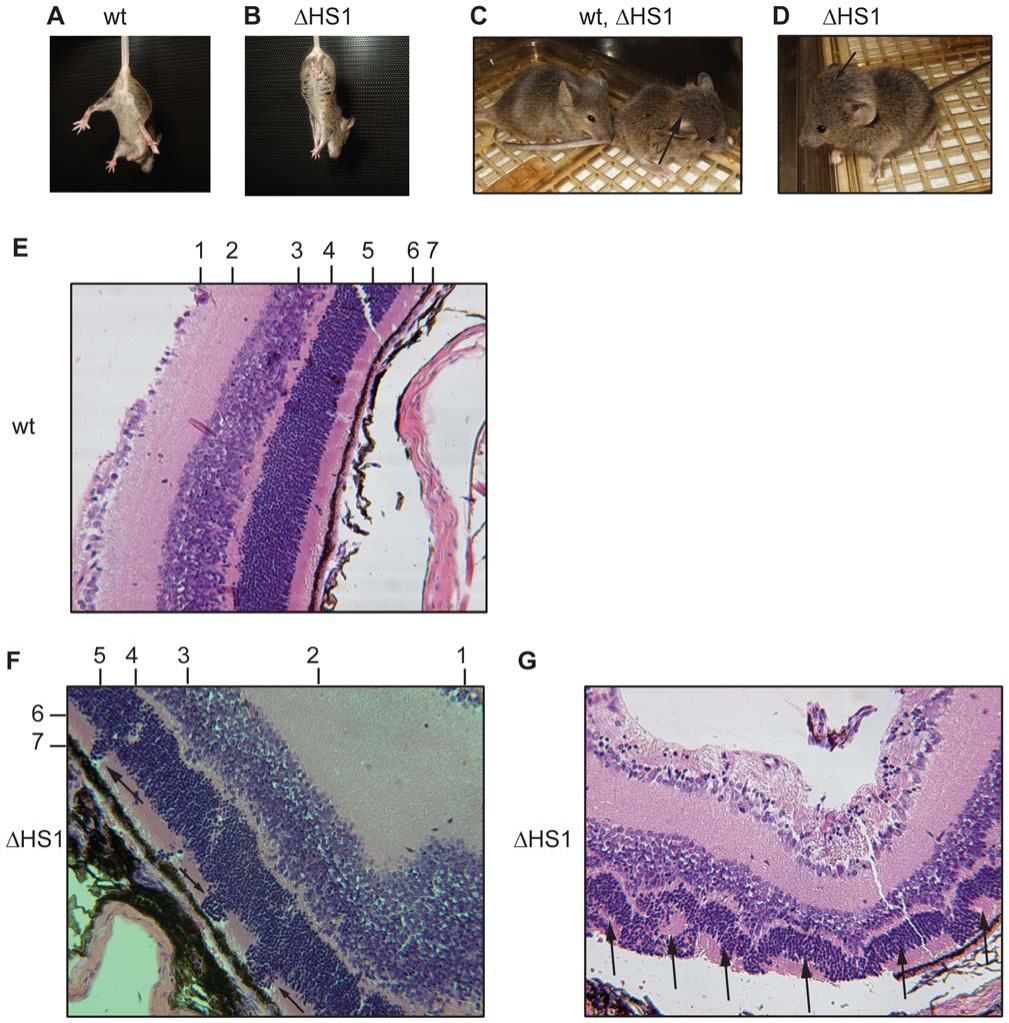
Complex phenotypes of homozygous *ΔHS1* mice. Homozygous *ΔHS1* mice exhibit an abnormal limp grasping phenotype (B) whereas wildtype (wt) mice do not (A). *ΔHS1* mice can develop severe hydrocephalus as indicated by arrows in (C) and (D). A wildtype mouse without hydrocephalus is shown in (C). The wildtype retina is organized in distinct layers (E): Stratum opticum and ganglionic layer (1), inner plexiform layer (2), inner nuclear layer (3), outer plexiform layer (4), outer nuclear layer (5), layer of rods and cones (6), pigment layer (7). The retina of homozygous *ΔHS1* mice shows external nuclei from the outer nuclear layer (5) in the layer of rods and cones (6) indicated by arrows in (F), or rosette formation of the outer nuclear layer (5) indicated by arrows in (G).

We performed targeted deletion experiments of the 5′IgH DNaseI hypersensitive sites to test their suggested function in IgH locus regulation. So far no major IgH related phenotype was identified. However, about 20% of homozygous *ΔHS1* mice develop a complex neurological phenotype and die at 3–5 weeks of age, likely due to a lack of food intake. These mice exhibit an abnormal limp grasping phenotype, i.e. mice clasp their front and hind feet almost immediately upon being lifted by their tail (Fig. 6A, B). Furthermore these mice develop a hydrocephalus, which is already visible at about one week of age and is enlarged over the following weeks (Fig. 6C, D). Histological analysis confirmed the presence of a hydrocephalus, revealed abnormal hindbrain development, and revealed retinal abnormalities (Fig. 6E, F, G). The wildtype retina is organized in a delicate layer system (Fig. 6E): stratum opticum and ganglionic layer (1), inner plexiform layer (2), inner nuclear layer (3), outer plexiform layer (4), outer nuclear layer (5), layer of rods and cones (6), pigment layer (7). In the *ΔHS1* mutant mice, the organization of retinal layers is impaired in such a way that nuclei from the outer nuclear layer are aberrantly located in the layer of rods and cones (Fig. 6F). In some more severe cases rosette formation in the outer nuclear layer is evident (Fig. 6G). Currently, we do not know what causes these phenotypes, but we exclude that this phenotype is caused by a second integration of the targeting vector at an undefined site in the genome (Figure S1). The deletion in the *ΔHS1* allele deletes 340 bp within intron 1 of *Zfp386*. Therefore, misregulation of that poorly described gene might cause the described phenotypes although other possibilities are conceivable.

## Discussion

This study aimed for elucidating the potential regulatory functions of a cluster of recently described DNaseI hypersensitive sites at the 5′ end of the IgH locus [9]. We performed targeted deletion of either the pro-B cell specific site HS1 (*ΔHS1*) or deletion of the entire cluster of hypersensitive sites (*ΔHSs*) in mice or in their lymphocytes, respectively. A potential regulatory element at the 5′end of the IgH locus was speculated to regulate processes such as IgH allelic exclusion, V_H_ germline transcription, differential accessibility or usage of distal versus proximal V_H_ gene families. Furthermore, it was suggested that the 5′end of the IgH locus might play a role in positioning the IgH locus in distinct subnuclear compartments [16], [17], [18], and it was suggested to harbor insulator or boundary capacity [19].

B- and T-lymphocytes homozygous for the *ΔHS1*, *RHS1*, and *ΔHSs* alleles appear to proceed through lymphocyte development in an unimpaired way. Data from RDBC chimeras generated from heterozygous *ΔHS1*, *RHS1*, and *ΔHSs* ES cells indicated that allelic exclusion is not affected in mutant B-cells and that mutant IgH alleles can undergo efficient V(D)J recombination of their IgH locus. Furthermore, data from PCR assays to analyze V(D)J recombination efficiency in mice with HS1 deleted on both alleles supports the notion that HS1 is not necessary for neither the D to J_H_ nor the V_H_ to DJ_H_ recombination step. Both proximal and distal V_H_ families as well as the distal most V_H_ segment V_H_J558.55 rearrange as efficiently as on wildtype alleles. Similarly, IgL loci in HS1 deleted B-cells rearrange at the same efficiency as wildtype IgL loci. Analysis of IgH V(D)J rearrangement status in hybridomas generated from heterozygous *ΔHS1*, *RHS1*, and *ΔHSs* B-cells also strengthens the idea that the deleted DNAseI hypersensitive sites would not regulate IgH V(D)J recombination. We tested for potential alterations associated with DNA end processing during V(D)J recombination by examining the CDR3 sequence obtained from homozygous *ΔHS1* B cells and found a distribution in length that was similar to wildtype B cells [20] (Figure S2).

We tested a potential effect of the cluster of DNaseI hypersensitive site on the process of IgH CSR. Assaying class switching upon different in vitro stimulations in wildtype and homozygous *Δ*HSs B-cells let us conclude that the cluster of 5′IgH DNaseI hypersensitive sites does not play a crucial role in CSR.

The only observed phenotypes so far occurred in homozygous *ΔHS1* mice and seem to be independent of the IgH locus. *ΔHS1* mice show abnormal limp grasping indicating a neurological abnormality, *ΔHS1* mice can develop severe hydrocephalus and exhibit retinal impairments. A possible explanation for these phenotypes is a potential defect in regulation of the zinc finger protein *Zfp386*. *ΔHS1* deletes a 340 bp region from intron 1 of *Zfp386* which might result in different splice forms, impaired expression levels, or expression patterns of this gene.

Overall, our analysis of the deletion of the pro-B cell specific site HS1 or the whole cluster of 5′IgH DNaseI hypersensitive sites did not support the existence of a cis-regulatory function of these elements regarding the IgH locus.

## Supporting Information

Figure S1 Single integration of the RHS1 targeting vector. The targeting vector (targeting vector RHS1), the targeted locus (RHS1), and the wildtype (wt) IgH locus with its 5′ flanking region are shown. VH, DH, JH indicate representative IgH V, D, and J segments. Exons 1, 2, and 3 of Zfp386 are shown as grey rectangles, DNaseI hypersensitive sites HS1, HS2, HS3a, and HS3b are shown as black ovals, the NeoR specific Southern probe as a black rectangle. X - XbaI. Southern analysis of XbaI digested genomic DNA from the targeted RHS1 clones 5 (lane 1) and 23 (lane 2) utilizing the NeoR specific probe shows a single 16.0 kb band. No bands are visible from untargeted wildtype ES cell DNA (lane 3). M - Fermentas 1 kb ladder.
(0.17 MB TIF)

Figure S2 CDR3 length in *ΔHS1* B cells. Peripheral B cells were isolated from one *ΔHS1* mouse and a wildtype mouse and amplified for V558-JH4 rearrangements. Heavy Chain CDR3 lengths were calculated as the number of nucleotides between the consensus Cys residue and the Trp residue. 23 individual sequences were analyzed from *ΔHS1* B cells and 10 from wildtype.
(0.05 MB PDF)

Table S1 (0.08 MB DOCX)
