# Supplementary figures and images for: Analysis of Mice Lacking DNaseI Hypersensitive Sites at the 5′ End of the IgH Locus
Source: PLoS One. 2010 Nov 15;5(11):e13992. doi: 10.1371/journal.pone.0013992 (PMC2981565; doi:10.1371/journal.pone.0013992)

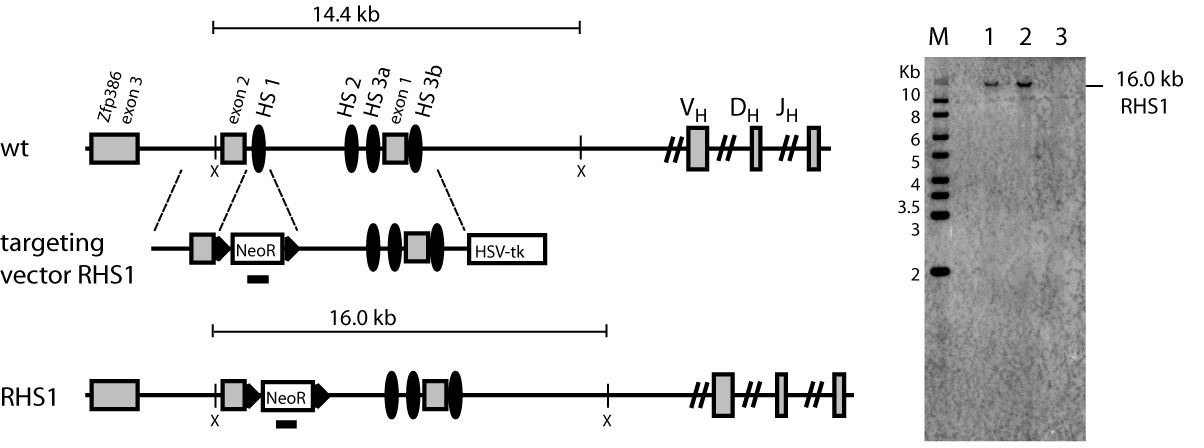

Supplement: Figure S1 — Single integration of the RHS1 targeting vector. The targeting vector (targeting vector RHS1), the targeted locus (RHS1), and the wildtype (wt) IgH locus with its 5′ flanking region are shown. VH, DH, JH indicate representative IgH V, D, and J segments. Exons 1, 2, and 3 of Zfp386 are shown as grey rectangles, DNaseI hypersensitive sites HS1, HS2, HS3a, and HS3b are shown as black ovals, the NeoR specific Southern probe as a black rectangle. X - XbaI. Southern analysis of XbaI digested genomic DNA from the targeted RHS1 clones 5 (lane 1) and 23 (lane 2) utilizing the NeoR specific probe shows a single 16.0 kb band. No bands are visible from untargeted wildtype ES cell DNA (lane 3). M - Fermentas 1 kb ladder. (0.17 MB TIF) [file pone.0013992.s001.tif]

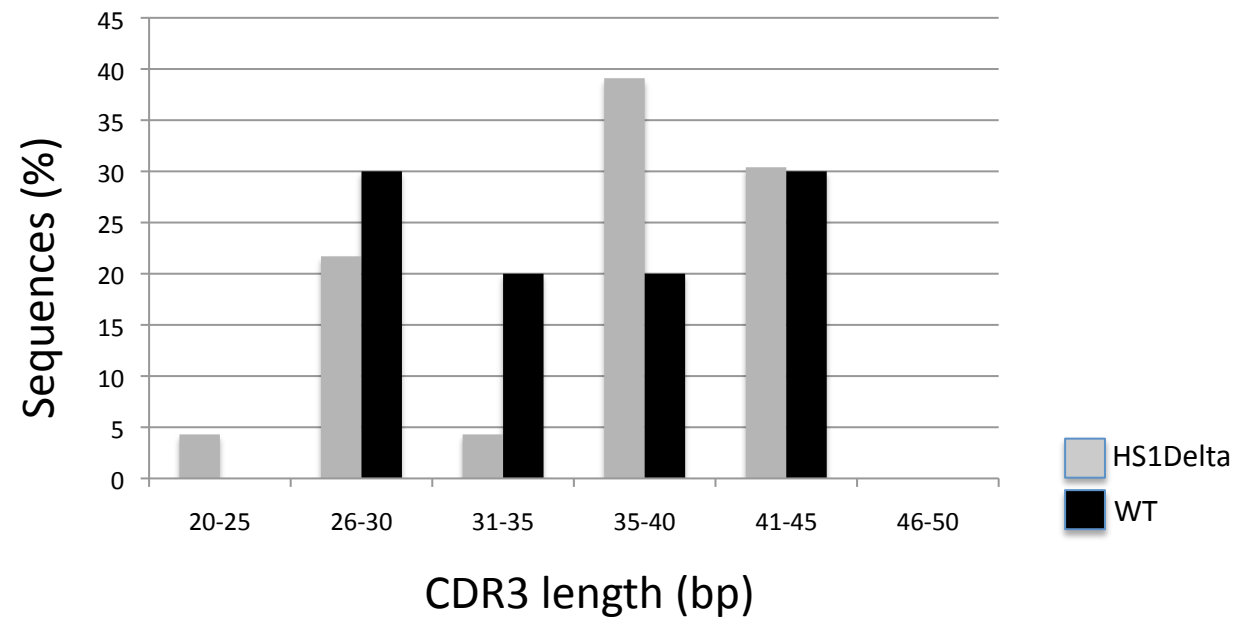

Supplement: Figure S2 — CDR3 length in ΔHS1 B cells. Peripheral B cells were isolated from one ΔHS1 mouse and a wildtype mouse and amplified for V558-JH4 rearrangements. Heavy Chain CDR3 lengths were calculated as the number of nucleotides between the consensus Cys residue and the Trp residue. 23 individual sequences were analyzed from ΔHS1 B cells and 10 from wildtype. (0.05 MB PDF) [file pone.0013992.s002.pdf]
