# Supplementary material for: Analysis of Mice Lacking DNaseI Hypersensitive Sites at the 5′ End of the IgH Locus
Source: PLoS One. 2010 Nov 15;5(11):e13992. doi: 10.1371/journal.pone.0013992 (PMC2981565; doi:10.1371/journal.pone.0013992)
Supplement: Table S1 — (0.08 MB DOCX) [file pone.0013992.s003.docx]

**Table S1. Oligonucleotide sequences and annealing temperatures in °C.**

RT- PCR-Primers: °C

V_H_J558 GLT For ATGGGATGGAGCTGGATCTT 55

V_H_J558 GLT Rev GACACACTCAGGATGTGTTTGTAG 55

β-Actin For TGGAATCCTGTGGCATCCATGAAAC 56

β-Actin Rev TAAAACGCAGCTCAGTAACAGTCCG 56

V(D)J recombination - PCR-Primers: °C

DQ52 For CCACAGG CTCGAGAACTTTAGCG 60

D_H_L For GGAATTCGMTTTTTGTSAAGGGATCTACTACTGTG 60

V_H_7183 For GTGGAGTCTGGGGGAGGCTTA 60

V_H_J558 For ARGCCTGGGRCTTCAGTGAAG 60

V_H_J558.55 For GAGGTCTGGACATACACTCATGTG 60

J_H_4 Rev AGGCTCTGAGATCCCTAGACAG 60

J_H_ probe CTATGGACTACTGGGGTCAAGGAA probe

Vκ For GGCTGCAGSTTCAGTGGCAGTGGRTCWGGRAC 60

Jκ Rev ATGCGACGTCAACTGATAATGAGCCCTCTCC 60

Jκ probe ACCAAGCTGGAGCTGAAACGTAAGTACAC probe

DLG5 For AAGTCTCAGGCTGGCCATTA 60

DLG5 Rev TTTACCACCCCTGACACACA 60
